# Depinning of domain walls in permalloy nanowires with asymmetric notches

**DOI:** 10.1038/srep32617

**Published:** 2016-09-07

**Authors:** Y. Gao, B. You, X. Z. Ruan, M. Y. Liu, H. L. Yang, Q. F. Zhan, Z. Li, N. Lei, W. S. Zhao, D. F. Pan, J. G. Wan, J. Wu, H. Q. Tu, J. Wang, W. Zhang, Y. B. Xu, J. Du

**Affiliations:** 1 National Laboratory of Solid State Microstructures and Department of Physics, Nanjing University, Nanjing 210093, P. R. China; 2 Collaborative Innovation Center of Advanced Microstructures, Nanjing 210093, P. R. China; 3 School of Electronic Science and Engineering, Nanjing University, Nanjing 210046, P. R. China; 4 Key Laboratory of Magnetic Materials and Devices & Zhejiang Province Key Laboratory of Magnetic Materials and Application Technology, Ningbo Institute of Material Technology and Engineering, Chinese Academy of Sciences, Ningbo, Zhejiang 315201, P. R. China; 5 Fert Beijing Institute, Beihang University, Beijing, P. R. China; 6 School of Electronic and Information Engineering, Beihang University, Beijing, China; 7 Department of Physics, University of York, York YO10 5DD, United Kingdom

## Abstract

Effective control of the domain wall (DW) motion along the magnetic nanowires is of great importance for fundamental research and potential application in spintronic devices. In this work, a series of permalloy nanowires with an asymmetric notch in the middle were fabricated with only varying the width (*d*) of the right arm from 200 nm to 1000 nm. The detailed pinning and depinning processes of DWs in these nanowires have been studied by using focused magneto-optic Kerr effect (FMOKE) magnetometer, magnetic force microscopy (MFM) and micromagnetic simulation. The experimental results unambiguously exhibit the presence of a DW pinned at the notch in a typical sample with *d* equal to 500 nm. At a certain range of 200 nm < *d* < 500 nm, both the experimental and simulated results show that the DW can maintain or change its chirality randomly during passing through the notch, resulting in two DW depinning fields. Those two depinning fields have opposite *d* dependences, which may be originated from different potential well/barrier generated by the asymmetric notch with varying *d*.

Due to its high density and non-volatility, racetrack memory (RM)^1^ is expected to be used in the next generation spintronic devices, in which magnetic domain walls (DWs) are utilized to store information. The position and motion of DWs can be controlled by creating artificial pinning sites such as constrictions or protrusions along the magnetic nanowires^2^,^3^. As the effect of DW contributing to magnetoresistance (DWMR)^4^,^5^ was considered to be an important phenomenon and might have wide applications in information storage devices, the mechanism of DWMR has also been intensively studied^5^,^6^,^7^,^8^,^9^,^10^. Those studies reported positive^6^,^7^,^8^ and negative^9^,^10^ DWMR effects due to spin-dependent scattering and anisotropic magnetoresistance (AMR), respectively. Generally speaking, the pinning and depinning processes of DWs can be modulated by external magnetic field directly^2^,^11^ or by spin-polarized current due to spin-transfer torque (STT) effect^1^,^8^,^10^,^12^,^13^. In order to better understand and control the pinning/depinning of DWs, various magnetic nanowire systems have been studied^2^,^4^,^10^,^14^,^15^. It was reported that the DW type and/or chirality, which depends on the detailed profile of the pinning sites, have significant effects on the depinning process of DWs in various magnetic nanowires^2^,^11^,^16^,^17^,^18^,^19^,^20^. Among them, creation of a well-defined single notch or a couple of notches along the nanowire is an efficient way to trap DW and control its movement.

Although there have many studies focused on the contribution from *symmetric* notches with various types and sizes^2^,^8^,^10^,^21^, the corresponding studies on *asymmetric* notches have rarely been carried out up to date. Very recently, W. Zhu *et al*.^11^ have observed two distinct depining fields by a high sensitive focused magneto-optical Kerr effect (FMOKE) magnetometer in a permalloy nanowire with an asymmetric notch. By micromagnetic simulation, they claimed that the different depinning fields are originated from the clockwise (CW) vortex DW (VDW) and the counter-clockwise (CCW) VDW, which are randomly generated in the nucleation stage and their chiralities keep unchanged during passing through the notch. Therefore, this stochastic phenomenon is due to the DW nucleation. Afterwards, J. Brandão *et al*.^22^ changed the outgoing angle *ϕ* with a fixed incoming angle *θ* (the definition of *ϕ* and *θ* can be referred to Fig. 1(b)) of the asymmetric notch in the permalloy nanowire to study the DW stochastic phenomenon. They assumed that the two chiralities of VDW are nucleated with equal probabilities at the nucleation pad and considered that the vortex chirality may change when the DW is passing through the notch, leading to the stochastic phenomenon. Therefore, two different depinning fields can be observed for the same nanowire and the stochastic phenomenon always existed in all the nanowires with *ϕ* varied from15° to 45°. Moreover, the occurrence probabilities of CW and CCW DWs and the corresponding depinning fields changed with varying *ϕ* in the same range.

For further understanding the underlying mechanism responsible for the stochastic phenomenon mentioned above, in this work, we *only* change the width (*d*) of the right arm of the permalloy nanowire with an asymmetric notch in the middle and studied the detailed pinning and depinning processes of DW. Note that the left arm, the length of the notch as well as the total length of the whole nanowire are all kept unchanged when *d* is changed. With decreasing *d*, an evolution from non-stochastic to stochastic phenomenon can be observed by FMOKE and also obtained by micromagnetic simulation. Those two different chiralities of DWs show distinctively different *d* dependent depinning fields, which are associated with different types of potential energies owing to the asymmetric notch with varied *d*.

## Results

Figure 2 show the magnetic hysteresis (*M*-*H*) loops obtained by FMOKE for the nanowires with *d* = 400 nm and 500 nm, respectively. The *M*-*H* loops for the other nanowires can be seen in Supplementary Material A. It needs to be emphasized that the *M*-*H* loop corresponds to the variation of the total magnetic moment of the nanowire versus the applied magnetic field (*H*_a_), which is different from that obtained from a small local area at the nanowire in refs 11,22. The formation of the *M*-*H* loops shown in Fig. 2 can be understood as follows. First of all, the magnetic moments of the nanowires are saturated in the positive direction under *H*_a_ = 320 Oe. For the nanowire with *d* = 400 nm, as shown in Fig. 2(a), when *H*_a_ is decreased to zero and begins to increase in the negative direction, two jumps appear at −26 Oe and −56 Oe, which indicates respectively that a DW is nucleated at the elliptic pad and pinned at the notch after its injection into the left arm and propagation to the notch. When the magnitude of *H*_a_ is increased further until the nanowire is negatively saturated, another two jumps appear at −108 Oe and −226 Oe, which correspond to two distinctively different depinning fields of the DW. In contrast to the descending branch, there are only three jumps in the ascending branch. Apart from almost the same quantities of the two depinning fields (108 Oe and 229 Oe for the ascending branch), the DW nucleation and pinning processes seem to be overlapped, demonstrating only one jump at 38 Oe. For the nanowire with *d* = 500 nm, as shown in Fig. 2(b), there are two jumps in both the descending and ascending branches, which correspond to the nucleation (or pinning) field and the depinning field of DW. Similar two-jump phenomena can also be observed in the nanowire when *d* is increased from 600 nm to 1000 nm (see Supplementary Material A), which indicates that the appearance of one or two depinning fields depends strongly on *d* and will be discussed in the latter parts. As for the overlap of the nucleation field and the pinning field appearing in the descending branch and/or the ascending branch, it may be caused by the following two reasons. Firstly, from the simulation results (will be shown later), it can be seen that these two fields are approaching, which may make them not easily to be distinguished in experiments. Secondly, because of structural defects, edge/surface roughness and some other artificial effects, there may be some pinning sites in the nanowire, which results in some difference in the initial magnetization reversal process between the descending and ascending branches. However, because the depinning field is much larger than the nucleation (or pinning) field of DW, these artificial effects play little role and the quantities of the depinning fields are nearly the same in the descending and ascending branches.

To confirm that the DW does exist in the nanowire and can be pinned at the notch, magnetic force microscopy (MFM) was operated at room temperature on the nanowire with *d* = 500 nm. To our knowledge, MFM can provide direct image of the stray field distribution along the nanowire with a fine tip magnetized perpendicular to the film plane^1^,^4^,^6^,^14^,^23^. Figure 3(a,a’) show the MFM images for the nanowire at remanence state after a positive saturation field was applied and removed shortly. These two images were obtained respectively by two oppositely magnetized tips. Due to the roughness and defects, there still exists stray field caused by the net magnetic moments around the notch^23^. In Fig. 3(a), the contrasts for the left-side and right-side of the notch are dark and bright, respectively^23^. However, if the tip is magnetized oppositely, the relative contrasts around the notch are exactly reversed, as clearly shown in Fig. 3(a’). Moreover, the contrast of the right end is dark in Fig. 3(a) and switches to bright in Fig. 3(a’). All these results demonstrate that the nanowire resembles two bar magnets lined together with different poles on both sides of the notch^23^, as indicated by the two blue arrows in Fig. 3(a,a’). According to the FMOKE results in Fig. 2(b) and the corresponding discussion, if a reversal field is applied at the plateau (between −70 Oe and −160 Oe), a DW will be pinned at the notch which is injected from the elliptic pad, and meantime the magnetic moments of the right arm have not reversed yet. To prove these statements by MFM, firstly the nanowire was saturated in the positive direction. After that, a reversal field of *H*_a_ = −150 Oe was applied and removed shortly. Then the MFM images were recorded as shown in Fig. 3(b,b’). Note that the magnetized states for the MFM tip in Fig. 3(a,b) (or Fig. 3(a’’)) are identical. From the results shown in Fig. 3(b,b’), the contrasts for the left side and the right side around the notch are nearly the same, which indicates that the magnetic moments of the left arm have reversed while those of the right arm remain unchanged. Therefore, now the nanowire resembles two bar magnets lined together with the same poles on both sides of the notch. In a word, the MFM images in Fig. 3 verify that a DW can be pinned around the notch by applying an external magnetic field properly.

In order to visualize the DW type and understand the detailed processes of DW pinning and depinning, micromagnetic simulation was performed. Note that the simulation was conducted on the nanowire *identical* to that in experiment (see details in the ‘Methods’ part), which favors to provide more reliable comparison between the simulated and experimental results. This is quite different from those done in refs 11,22, where the micromagnetic simulation is conducted on a part of the nanowire around the notch [11] or a much shortened nanowire [22] because the nanowires studied in experiments are too long (tens of or about 100 micronmeters in length). The detailed reversal process for the permalloy nanowire with *d* = 500 nm is simulated and exhibited in Fig. 4(a) with the magnitude of *H*_a_ increased from zero to 400 Oe which can fully saturate the nanowire’s magnetic moments. Several specific points marked as N, P, D in this plot represent the initial state of the DW nucleation, pining and depinning process, respectively. Moreover, P’ means the final state of the pinning process, after which the depinning process begins.

Figure 4(b) show the magnetic moment distribution corresponding to those specific points marked in Fig. 4(a), in which the arrows combined with the contrasts indicate the orientations of the magnetic moments along the nanowire. When *H*_a_ is zero, almost all the magnetic moments orient along the positive direction except that some portions are parallel to the edges due to strong shape anisotropy^3^,^21^. When |*H*_a_| is increased, the magnetic moments of the ellipse part begin to decrease with a first jump at *H*_a_ = −22.5 Oe, which means a CW VDW nucleating at point N (Picture N). Although the simulation results show that the probabilities of CW and CCW VDWs nucleated at the elliptic pad are equal, only CW VDW is taken as an example to be considered here and in the latter parts of the paper. When *H*_a_ reaches −47.5 Oe, the CW VDW is injected into the left arm and trapped at the notch^11^,^22^,^24^ with a second jump accompanied by a significant reversal of the magnetic moments, which means the magnetic moments of the left arm have almost reversed to the negative direction while those of the right arm still keep unchanged (Picture P). Note that the difference between the nucleation and pinning fields is only 25 Oe, which may be too small to be distinguished by the *M*-*H* loop measurement. From the enlarged view of the notch area in Picture P, one can find that the magnetic moments near the left edge of the notch still point to the original direction due to strong shape anisotropy. Moreover, the CW VDW contracts and the core of the CW vortex moves to the upper corner of the notch left-side. If the applied field is removed now, the CW VDW will be expanded. To prove this statement, a simulation is performed at remanence state after *H*_a_ = −100 Oe is applied and then removed shortly, a larger CW VDW will be formed, as shown by the enlarged view in Fig. 4(c). This picture is in good agreement with the MFM images (Fig. 3(b,b’)) obtained at remanence state.

With further increase of |*H*_a_| above 47.5 Oe, the CW VDW will move rightwards with size shrinkage, as displayed by the comparison between pictures P and P’. When *H*_a_ reaches −157.5 Oe, the total magnetic moments of the right arm reverse finally with the DW depinning (Picture D) and a third jump occurs in Fig. 4(a). This depinning field value is perfectly consistent with that (−160 Oe) observed by FMOKE as displayed in Fig. 2(b). In addition, for the nanowires with *d* increased from 500 nm to 1000 nm, this consistency is always kept well, which will be addressed in the latter parts. As for the inconsistency of the nucleation and pinning fields between the experimental and simulated results, it may be caused by some artificial effects as mentioned previously. Finally, as for the processes of DW reaching and passing through the notch, some transformation and even the chirality change of the DW may take place due to interacting with the notch (see details in Supplementary Material B), which depends strongly on *d* and will be discussed in the following.

In order to investigate the effect of the asymmetric notch shape on the pinning/depinning behavior of DW, further simulation was performed with increasing *d* from 200 nm to 1000 nm with an increment of 100 nm, which are the same as those done in experiments. To aid our understanding, the reversal process of the single right arm without the left part is also simulated. Figure 5(a) shows the *d* dependences of the nucleation field (*H*_N_), pinning field (*H*_P_), depinning field (*H*_D_) of DW and reversal field of the single arm (*H*_R_). Due to unchanged left part of the nanowire, it is reasonable that the values of *H*_N_ and *H*_P_ are kept respectively at −22.5 Oe and −47.5 Oe with varying *d*. In contrast, the magnitude of *H*_R_ increases quickly with decreasing *d* due to significant enhancement of shape anisotropy. The most important and interesting result in Fig. 5(a) is the *d* dependence of *H*_D_. When *d* decreases gradually from 1000 nm, the CW DW maintain its chirality around the notch and meantime *H*_D_ increases slowly in magnitude and becomes stable at −157.5 Oe when *d* reaches about 700 nm. When *d* decreases further from 500 nm to above 200 nm (e.g. 400 nm), the DW either maintains or changes its chirality during passing through the notch, leading to two values of *H*_D_, as indicated by the dash-lined box in the inset of Fig. 5(a). Finally, when *d* deceases to be very small (e.g. 200 nm), only CCW DW appears with one *H*_D_ again. The simulations for the same nanowire were repeated fifteen times to confirm the reliability of the results. For example, all the fifteen simulations show that only one chirality of the DW can exist at the notch for the nanowire with *d* = 500 nm. Finally, it needs to be noted that the values of the experimental *H*_D_ (indicated by ‘star’ dots) obtained by FMOKE are in good consistent with those of simulation when *d* decreases from 1000 nm to 500 nm. However, for the nanowire with *d* = 400 nm, the split of the two *H*_D_ values in experiment are much more significant than that in simulation. This may be due to structural defects, edge/surface roughness^25^,^26^, which are not included in the micromagnetic simulation^11^. When *d* is less than 400 nm, the depinning process can hardly be detected due to the sensitivity limitation of the FMOKE.

## Discussion

The above calculated *d* dependence of *H*_D_ can be understood as follows. When *d* decreases from 100 nm to 700 nm, the CW VDW maintains its chirality and the core of the DW is trapped at the upper part of the notch left-side (see picture P in Fig. 4(b)), where the DW magnetization points in the same direction as that of the notch edge. Therefore, the DW has to overcome the attraction (side well potential^2^,^16^) to travel further. Hence, *H*_D_ (for CW VDW) is determined by two factors which are the potential energy of the side well (*E*_W_) and the shape anisotropy energy of the right arm (*E*_A_). When 700 nm ≤ *d *≤ 1000 nm, *E*_A_ is smaller than *E*_W_ and thus *H*_D_ is determined by *H*_R_, leading to the two plots of *H*_R_ ~ *d* and *H*_D_ ~ *d* overlapped and |*H*_D_| increasing gradually from 100 Oe to 157.5 Oe with decreasing *d*. When *d* decreases further from 700 nm before it reaches 500 nm, *H*_D_ keeps at −157.5 Oe because *E*_W_ is smaller than *E*_A_ and kept nearly unchanged due to invariable notch left-side. If *d* is decreased further and still larger than 200 nm (e.g. *d* = 400 nm), the CW VDW will maintain or change its chirality during passing through the notch, resulting in two different depinning fields^2^,^11^,^16^,^17^,^22^. Note that |*H*_D_| for CCW VDW is always smaller than that for CW VDW, which is well consistent with previous results^11^,^16^,^22^. When *d* decreases to 200 nm, the stochastic phenomenon disappears again and only CCW VDW exists. These above results indicate that the energy for maintaining the chirality of CW DW is comparable to that for changing the chirality if 200 nm < *d* ≤ 400 nm whereas the latter becomes significantly smaller than the former if *d* is reduced to 200 nm. Figure 5(b) shows the spin structures of CW VDW (left panel) and CCW VDW (right panel) with *d* = 400 nm at state P’. Note that these two DWs are both transformed from the same spin structure of state P as shown in Fig. 4(b) and each of them is generated as a result of maintaining or changing the chirality of initial CW VDW (see details in Fig. S3 in Supplementary Material B). From the right panel in Fig. 5(b) one can see that the CCW VDW is trapped at the right side of the notch central part, where the DW magnetization points in the opposite direction as that of the notch edge. Therefore, the DW has to overcome the repulsion (central barrier potential^2^,^16^) to travel further. As *d* decreases from 400 nm to 200 nm, the angle between the direction for the magnetic moments of the notch right-side and the longitudinal-positive direction decreases. This will make the magnetic moments more difficult to reverse towards left due to enhancement of the potential energy of the central barrier (*E*_B_), leading to |*H*_D_| of CCW VDW increasing with decreasing *d* accordingly. On the other hand, although the geometry of the notch left-side doesn’t change with *d, E*_W_ may be still lowered a little, leading to *H*_D_ of CW VDW decreasing slightly with decreasing *d* in the same *d* region. Therefore, the two different types of DWs have opposite *d* dependences of *H*_D_ at a certain small-*d* range.

Previous studies^22^ have reported that the stochastic phenomenon always exists with the outgoing angle *ϕ* ranged from 15° to 45°, leading to two depinning fields. In this presented work, the left arm including the elliptic pad, the lengths of the notch and the total nanowire are all maintained while only the width of the right arm is decreased from 1000 nm to 200 nm. Although the angle of *ϕ* is still restricted to the range between 15° to 45° for 500 nm < *d* ≤ 1000 nm, the stochastic phenomenon does not exist anymore. In our samples, the stochastic phenomenon can only exist in a very narrow range of 200 nm < *d* ≤ 400 nm. Therefore, the width of the arm at the right side of the asymmetric notch may be a crucial parameter determining the existence of the stochastic phenomenon, which may help us to design DW-based spintronic devices avoiding this phenomenon from the view point of application at present.

In summary, the detailed processes of DW pinning and depinning in permalloy nanowire with an asymmetric notch have been studied both experimentally and by micromagnetic simulation when *d* changes from 1000 nm to 200 nm. FMOKE and MFM techniques unambiguously verify that the DW can be trapped at the notch in a representative nanowire with *d* = 500 nm. The studies of FMOKE and micromagnetic simulation reveal that the DW can maintain or change its chirality randomly during passing through the notch at a certain small-*d* range (e.g. *d* = 400 nm), resulting in two depinning fields. These results can be understood qualitatively by the *d* dependent potential energy of the side well/central barrier rooted from the shape-varied notch. These findings will be potentially useful in designing new generation DW-based spintronic devices.

## Methods

### Sample design, fabrication and the *M*-*H* loop measurement

**Figure 1:**
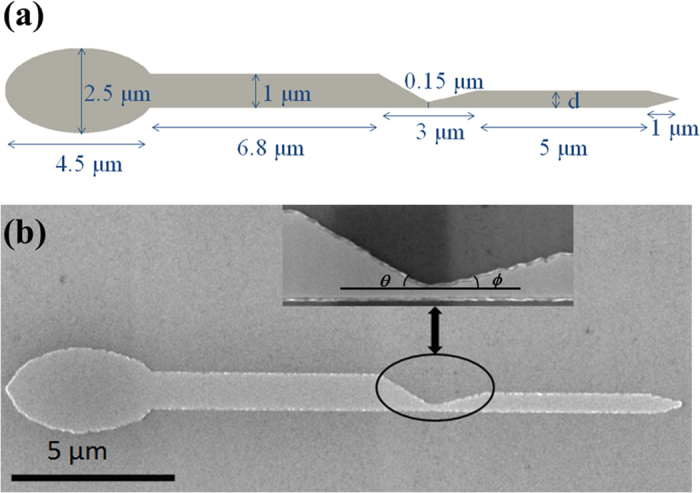
(**a**) Schematic illustration of the permalloy nanowire designed for experiment and simulation. (**b**) SEM image of the fabricated permalloy nanowire with *d* = 500 nm and the enlarged view around the notch. The black circle and the double-headed arrow are guides to eyes.

**Figure 2:**
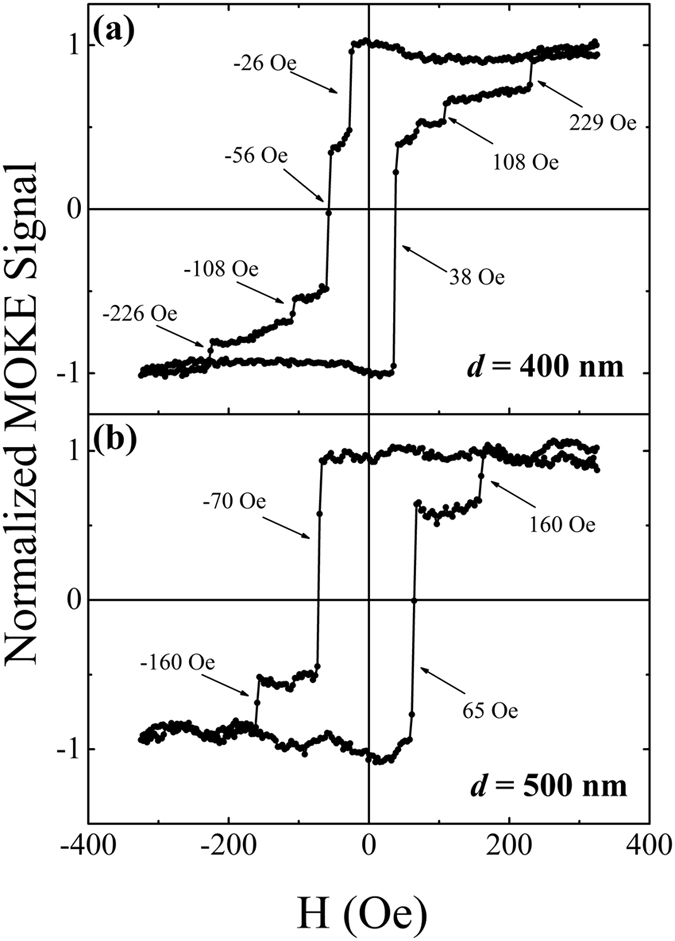
The *M*-*H* loops obtained by FMOKE for the nanowires with. (**a**) *d* = 400 nm and (**b**) *d* = 500 nm, respectively.

**Figure 3:**
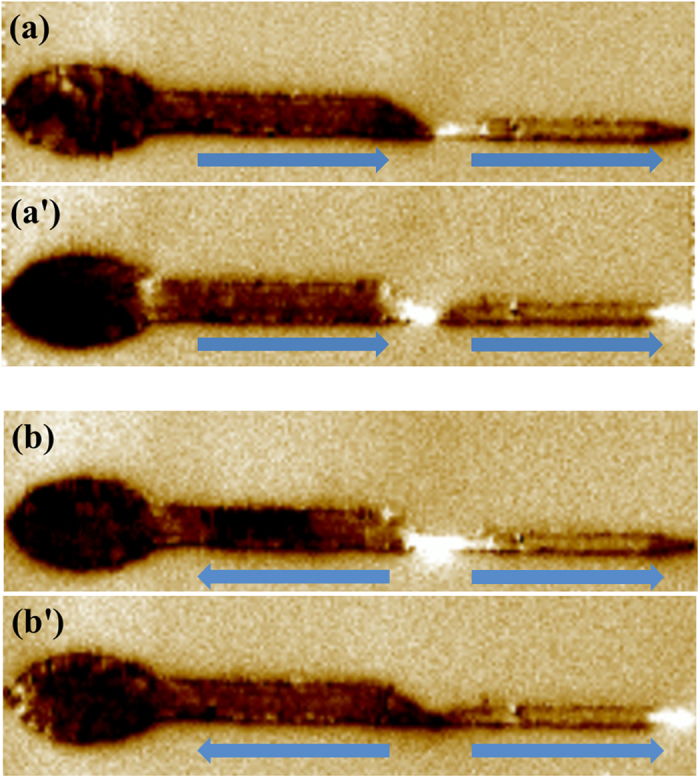
MFM images obtained at zero field after saturation in the positive direction. ((**a**,**a’**)), and after applying a reversal field of −150 Oe ((**b**,**b’**)) by using a tip at two oppositely magnetized states. The two blue arrows in each graph indicate the magnetic moment directions for the left and right arms, respectively.

**Figure 4:**
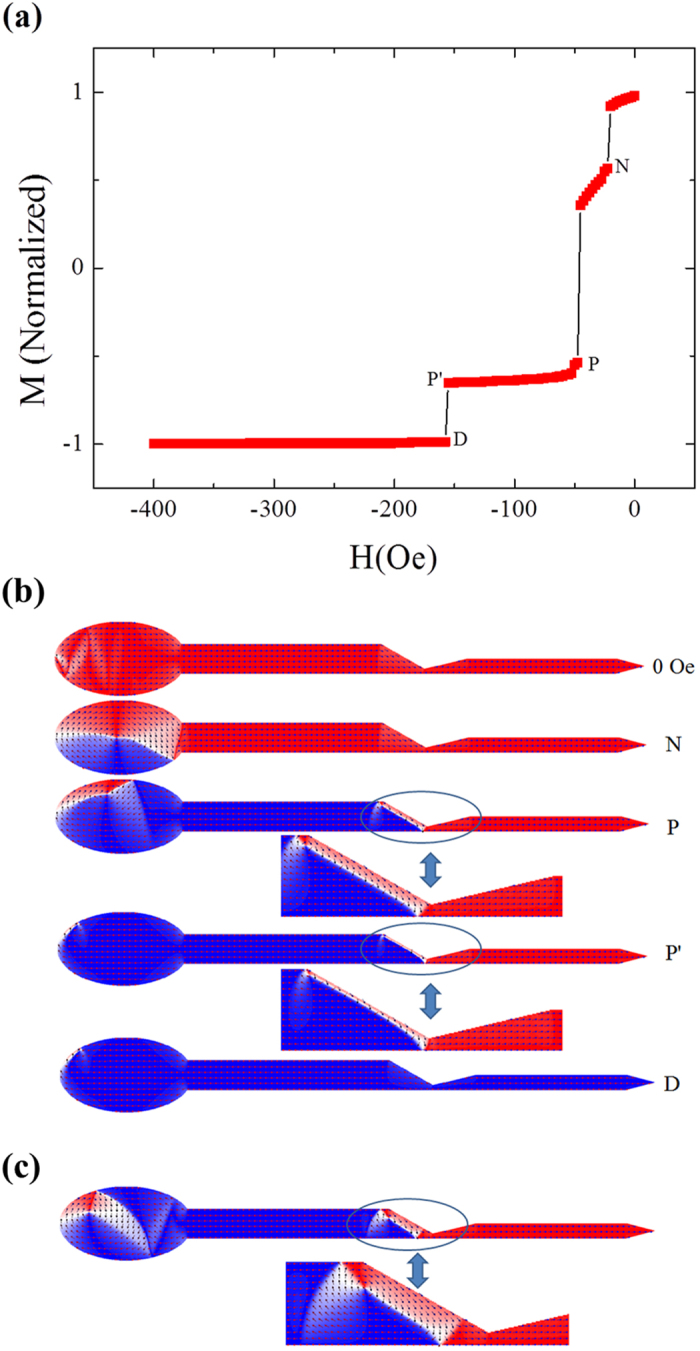
The micromagnetic simulation results on the permalloy nanowire with *d* = 500 nm for. (**a**) the total magnetic moments against *H*_a_ varied from 0 to −400 Oe after saturation in the positive direction, (**b**) the schematic illustrations of the magnetic moment distribution at several states marked in (**a**,**c**) the spin structure of CW VDW at zero field. The blue circles and the double-headed arrows are guides to eyes.

**Figure 5:**
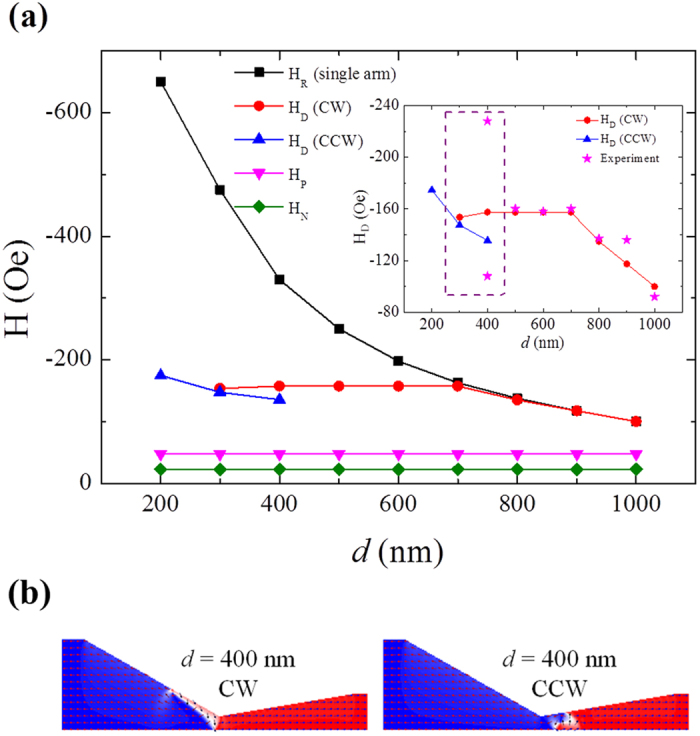
(**a**) The calculated *d* dependences of the nucleation field (*H*_N_), the pinning field (*H*_P_), the depinning field (*H*_D_) of DWs and the reversal field of the single arm (*H*_R_). The inset is the enlarged view of the *d* dependent depinning fields of CW VDW and CCW VDW, and the star dots denote the experiment results. (**b**) The calculated spin structure of the CW and CCW DWs around the notch at state P’ for the nanowire with *d* = 400 nm.

The schematic illustration for the designed magnetic nanowire is shown in Fig. 1(a). The total length of the nanowire is about 20 μm. The left end is a large ellipse for domain nucleation and injection while the right end is a tip to prevent formation of undesired DWs^11^,^27^. There is an asymmetric inward notch located in the middle of the nanowire. The length of the asymmetry notch is 3 μm and the distance between the bottom end of the notch and the edge of the nanowire is 0.15 μm. While the width of left arm is kept at 1 μm, the right arm width *d* is varied from 200 nm to 1000 nm with a step of 100 nm in both experiments and micromagnetic simulations. Note that although the outgoing angle *ϕ* also changes when *d* is varied, the length of the notch (3 μm) is kept unchanged, which is different from that in ref. 22. In experiments, a series of permalloy (Fe_20_Ni_80_) nanowires were fabricated on the thermally oxidized Si substrate by electron-beam lithography followed by electron-beam evaporation and lift-off. Each nanowire contains a 25 nm thick FeNi layer and a 2 nm gold capping layer for anti-oxidation protection. The scanning electron microscope (SEM) image of the FeNi nanowire with *d* = 500 nm is shown in Fig. 1(b), in which the enlarged drawing of the notch is also exhibited. The geometry and the size of each part for the actual nanowire shown in Fig. 1(b) are comparable to the designed ones shown in Fig. 1(a) except that the edges of the nanowire have a little roughness associated with the fabrication process. In Fig. 1(b), the incoming angle and outgoing angle of the notch are denoted as *θ* and *ϕ*, respectively. As *d* is increased from 200 nm to 1000 nm, *θ* is held at ~29.5° while *ϕ* changes from ~1.9° to ~29. 5°.

The magnetic hysteresis (*M*-*H*) loops of different nanowires are recorded with the magnetic field applied in the film plane and parallel along the nanowire by a high sensitive FMOKE (NanoMOKE III, Durham Magneto Optics Ltd.) with a focused laser spot of 30 μm in diameter, which can cover all the regions of a single nanowire. Each loop is obtained by averaging 100~150 single-shot measurements to achieve greatly improved signal-to-noise ratio. This measurement protocol is repeated three times for the same nanowire and the results are almost the same, confirming the reliability of the measurement.

### Calculation details

OOMMF (the Object Oriented MicroMagnetic Framework) based on Landau-Lifshitz-Gilbert (LLG) equation was used to simulate the magnetization reversal processes of a series of 25 nm thick permalloy nanowires with several standard parameters (saturation magnetization *M*_S_ = 8.6 × 10^5^ A/m, exchange coefficient *A* = 1.3 × 10^−11^ J/m,Gilbert damping constant α = 0.01)^11^,^21^,^22^. Because the magneto-crystalline anisotropy of the patterned nanowire is very weak, the anisotropy energy constant *K* is regarded as 0 in the simulation. The cell size was chosen as 12.5 nm for reasonable simulation time. From the experimental results, we have known that there is no essential difference of the DW pinning/depinning process between the descending and ascending branches of an *M*-*H* loop. Therefore, in order to save the simulation time, only the negative reversal process after saturation in positive direction is simulated. Firstly, all the magnetic moments along the nanowire are set in the longitudinal-positive direction, which is equivalent to applying a saturation *H*_a_ in this direction. Then a negative *H*_a_ is applied to the nanowire and its reversal process is studied carefully with increasing the magnitude of *H*_a_ gradually.

## Additional Information

**How to cite this article**: Gao, Y. *et al*. Depinning of domain walls in permalloy nanowires with asymmetric notches. *Sci. Rep.* **6**, 32617; doi: 10.1038/srep32617 (2016).

## Supplementary Material

Supplementary Information
