# Supplementary material for: Depinning of domain walls in permalloy nanowires with asymmetric notches
Source: Sci Rep. 2016 Sep 7;6:32617. doi: 10.1038/srep32617 (PMC5013472; doi:10.1038/srep32617)
Supplement: Supplementary Information [file srep32617-s1.pdf]

Depinning of domain walls in permalloy nanowires with asymmetric notches

Y. Gao[1], B. You[1, 5, *], X. Z. Ruan[2], M. Y. Liu[2], H. L. Yang[3], Q. F. Zhan[3], Z. Li[6, 7], N. Lei[6, 7], W. S. Zhao[6, 7], D. F. Pan[1], J. G. Wan[1], J. Wu[4], H. Q. Tu[1], J. Wang[1], W. Zhang[1], Y. B. Xu[2], J. Du[1, 5, *]

[1]*National Laboratory of Solid State Microstructures and Department of Physics, Nanjing University, Nanjing 210093, P. R. China*

[2]*School of Electronic Science and Engineering, Nanjing University, Nanjing 210046, P. R. China*

[3]*Key Laboratory of Magnetic Materials and Devices & Zhejiang Province Key Laboratory of Magnetic Materials and Application Technology, Ningbo Institute of Material Technology and Engineering, Chinese Academy of Sciences, Ningbo, Zhejiang 315201, P. R. China.*

[4]*Department of Physics, University of York, York YO10 5DD, United Kingdom*

[5]*Collaborative Innovation Center of Advanced Microstructures, Nanjing 210093, P. R. China*

[6]*Fert Beijing Institute, Beihang University, Beijing, P. R. China*
[7]*School of Electronic and Information Engineering, Beihang University, Beijing, China*

*Authors to whom correspondence should be addressed. Electronic addresses: youbiao@nju.edu.cn and jdu@nju.edu.cn

SUPPLEMENTARY MATERIAL

A. *M-H* loops for the nanowires with various *d*

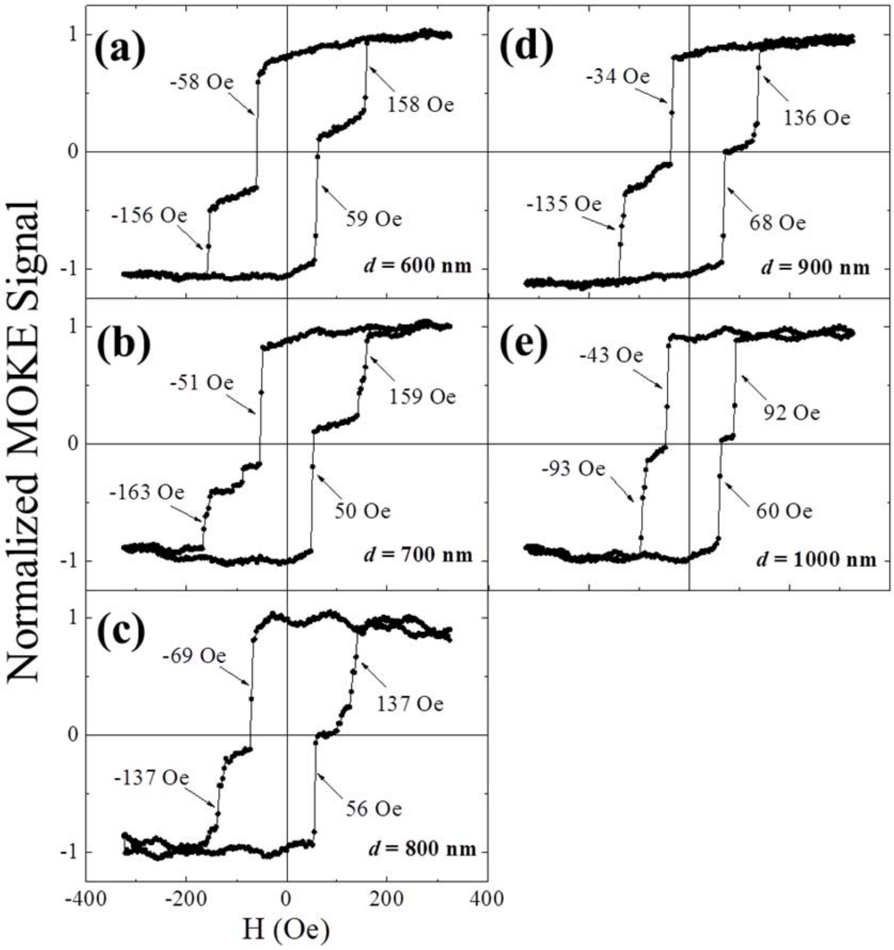

Figure S1 *M-H* loops obtained by FMOKE for the nanowires with *d* = 600 nm (a), 700 nm (b), 800 nm (c), 900 nm (d), 1000 nm (e).

A series of permalloy nanowires with *d* varied from 200 nm to 1000 nm were fabricated and FMOKE was utilized to record their *M-H* loops. When *d* is as small as 200 nm or 300 nm, due to the sensitivity limitation of the FMOKE, the relative *M-H* loop can hardly reflect the entire magnetization reversal process of the nanowire and therefore are not shown here. Since the results for *d* = 400 nm and 500 nm have already been shown in the manuscript, only the *M-H* loops with *d* varied from 600 nm to 1000 nm are exhibited in Fig. S1. As indicated by the arrows in Fig. S1, there are

two clear jumps or coercive fields for each sample. As described in the manuscript, the smaller coercive field may be the DW pinning field merged with the nucleation field due to introduction of some artificial effects during the sample fabrication process. Therefore, with increasing *d*, it does not present a clear variation trend. The larger coercive field is the DW depinning field. Similar to the result for *d* = 500 nm, there is also only one depinning field for each nanowire when *d* is increased from 600 nm to 1000 nm and it decreases with increasing *d* generally. It needs to be emphasized that both the variation trend of the DW depinning field versus *d* and their values are in good consistent with those obtained by the micromagnetic simulation, as clearly shown in the inset of Fig. 5(a) in the manuscript.

B. The processes of DW reaching and passing through the notch

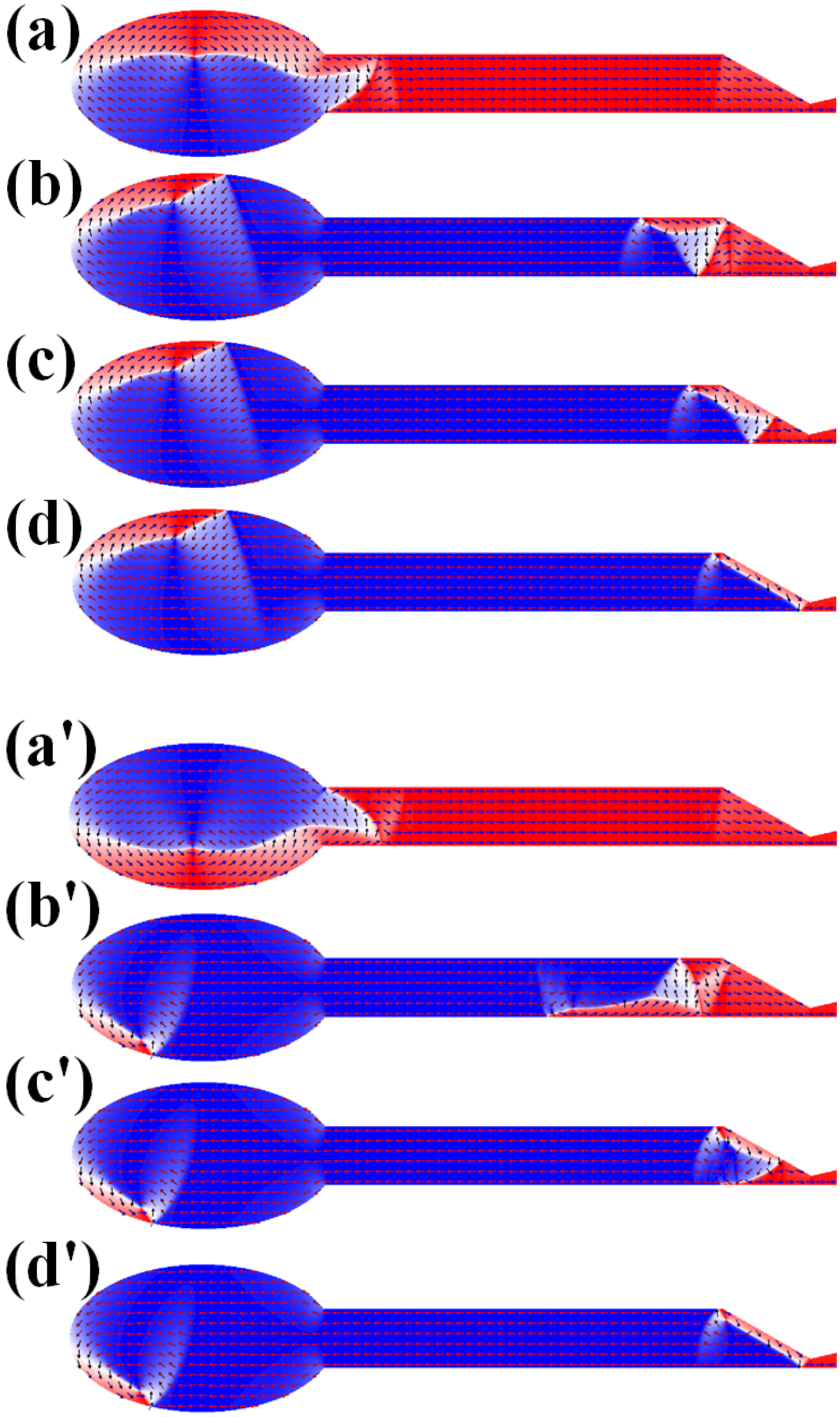

Figure S2 The processes of CW VDW ((a)-(d)) and CCW VDW ((a'-d')) nucleation, propagation along the left arm and pinning at the notch for the nanowire with d = 500 nm.

In this section we will discuss briefly how the DW reaches and passes through the notch when d is reduced. For all the nanowires addressed in the present work, the left arm including the elliptic pad and the notch left-side does not change at all, so the DW nucleation, propagation and reaching the notch should be identical. Taken the nanowire with d = 500 nm as a representative, several snapshots of the DW propagation and reaching the notch have been shown in Fig. S2. First, the CW VDW and CCW VDW are nucleated at the elliptic pad with equal probabilities. In the case of CW VDW nucleation, as displayed by the images from (a) to (d) in Fig. S2, after the CW VDW is injected into the left arm, it propagates along the nanowire and keeps its chirality till it reaches the notch left-side. Meantime, the core of the VDW moves upwards and finally the VDW will shrink into a transverse-like DW along the left edge of the notch. In the other case of CCW VDW nucleation, as displayed by the images from (a') to (d') in Fig. S2, a new CW VDW is nucleated near the notch during the processes of the CCW VDW propagating along the nanowire, which is resulted from strong shape anisotropy provided by the left edge of the notch. The core of the CCW VDW moves downwards and finally the CCW VDW will be annihilated. Meantime, the core of the CW VDW moves upwards and a transverse-like DW will be formed, similar to the final state of the CW VDW nucleation case. Therefore, despite of the chirality of the DW nucleated at the elliptic pad, the same type of DW will be formed and pinned at the notch left-side before it passes through the notch.

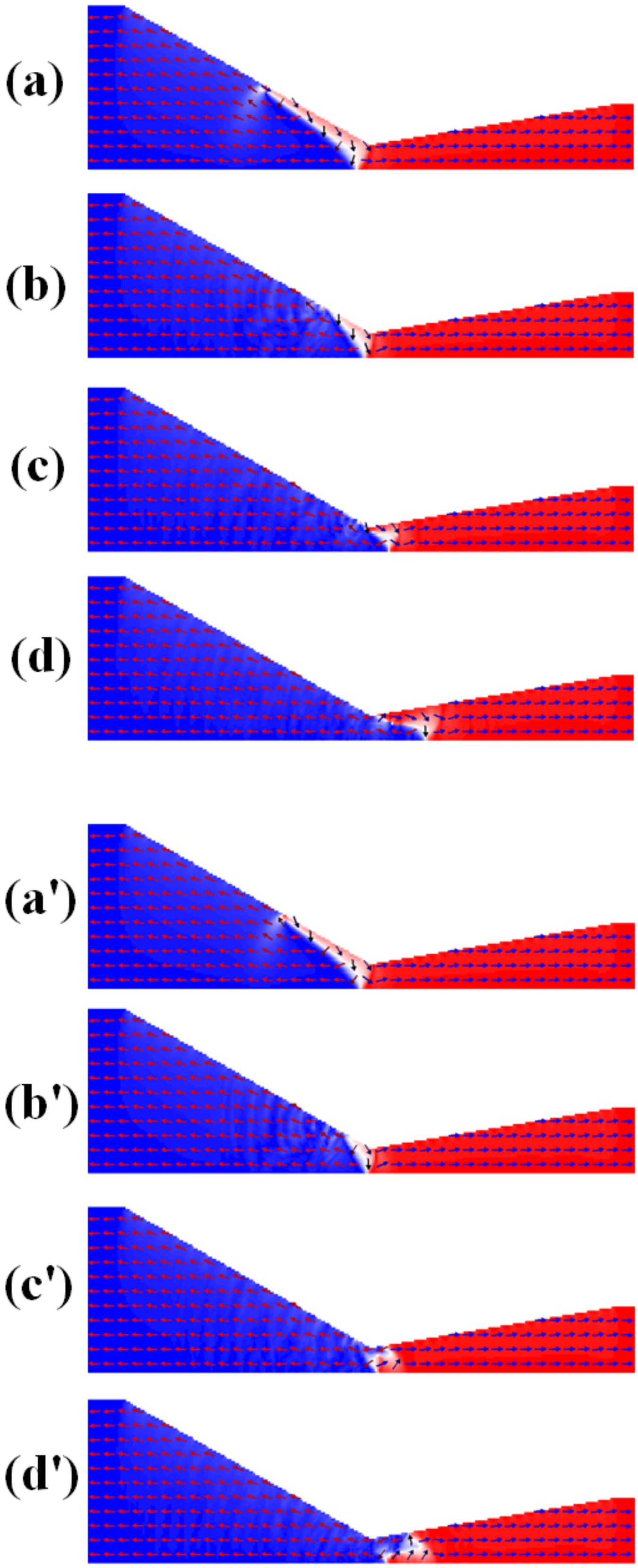

Figure S3 The processes of CW VDW maintaining ((a)-(d)) and changing ((a'-d')) its chirality when passing through the notch for the nanowire with d = 400 nm.

After the CW VDW (transverse-like) pinned at the notch left-side passes through the notch, its chirality will keep unchanged when 500 nm ≤ d ≤ 1000 nm or change randomly when 200 nm < d < 500 nm, which has been discussed in details in the manuscript. Figure S3 shows several snapshots which briefly describes the processes of the DW passing through the notch for the nanowire with d = 400 nm. First, the core of the CW VDW moves downwards along the left edge of the notch, and the size of the DW decreases simultaneously, as exhibited from image (a) to image (b) (or image (a') to image (b')). When the DW is passing through the narrowest part (apex) of the notch, it will maintain (see images (c) and (d)) or change its chirality (see images (c') and (d')). Afterwards, the DW is expanded and a complete CW VDW (image (d)) or CCW VDW (image (d')) will be formed at the notch right-side. These two VDWs with opposite chiralities result in two depinning fields. Therefore, the DW stochastic phenomenon in the present work is essentially come from the DW passing through the narrowest constriction in the nanowire.
